# Cavernous Hemangioma: A Rare Adrenal Tumor Associated With Hyperaldosteronism

**DOI:** 10.7759/cureus.79251

**Published:** 2025-02-18

**Authors:** Hannah Sage, Edward Jones, Vladimir Neychev

**Affiliations:** 1 Surgery, University of Central Florida College of Medicine, Orlando, USA; 2 Pathology, AdventHealth Orlando, Orlando, USA

**Keywords:** adrenal gland neoplasms, adrenal lesion, adrenal pheochromocytoma, adrenal surgery, cavernous hemangioma surgery

## Abstract

Adrenal cavernous hemangiomas are rare benign venous malformations characterized by vascular dysmorphogenesis. A 53-year-old male patient was referred for a surgical consultation with a 5 cm heterogeneous, lipid-poor, incidental left adrenal mass. It was detected on a computed tomography (CT) scan performed for left upper quadrant pain. His past medical history was significant for poorly-controlled hypertension on a multi-drug regimen, anxiety, headaches, insomnia, and palpitations. Laboratory testing performed by his primary care physician revealed elevated plasma catecholamines, which were concerning for pheochromocytoma. A repeat workup by endocrinology and endocrine surgery showed biochemical evidence of primary hyperaldosteronism with an aldosterone/plasma renin ratio of 36.8 and normal plasma catecholamines and metanephrines. Due to the biochemical workup, size, and radiological features of the mass, a decision to proceed with a left adrenalectomy was made. The patient was started on phenoxybenzamine two weeks before surgery due to a high index of clinical suspicion for a pheochromocytoma, despite the equivocal biochemical workup. Surgical pathology revealed a cavernous hemangioma with pseudonodular thickening of the adrenal cortex without evidence of malignancy, adrenal adenoma, or pheochromocytoma. On follow-up, the patient was on fewer antihypertensive medications with normal blood pressure and a normalized aldosterone/plasma renin ratio. A diagnosis of cavernous hemangioma should be considered for adrenal masses of uncertain biology and biochemical activity.

## Introduction

Adrenal cavernous hemangiomas are benign lesions composed of dysmorphic endothelial cells [1]. Due to their rarity, the literature primarily deals with case reports with approximately 70 cases published since the first adrenal cavernous hemangioma was surgically removed in 1955 [2,3]. There are no specific laboratory tests that can be used to diagnose cavernous hemangioma and imaging findings are also non-specific [4]. There have been few documented cases of hormonally active cavernous hemangiomas which can present similarly to other benign or malignant adrenal tumors [3].

We report a case of a large left adrenal cavernous hemangioma in a 53-year-old man with abdominal pain, poorly-controlled hypertension, anxiety, headaches, insomnia, and palpitations. This is among the few reported cases of possible aldosterone-overproducing adrenal hemangioma. 

## Case presentation

A 53-year-old male patient with a past medical history of hypertension on a multi-drug treatment regimen including amlodipine 10 mg daily, lisinopril 40 mg daily, and propranolol 25 mg twice a day, hyperlipidemia, and type II diabetes mellitus presented to his primary care physician (PCP) with a complaint of five years of intermittent, stabbing left lower abdominal and back pain. He also noted intermittent palpitations at night as well as anxiety, headaches, and insomnia.

**Figure 1:**
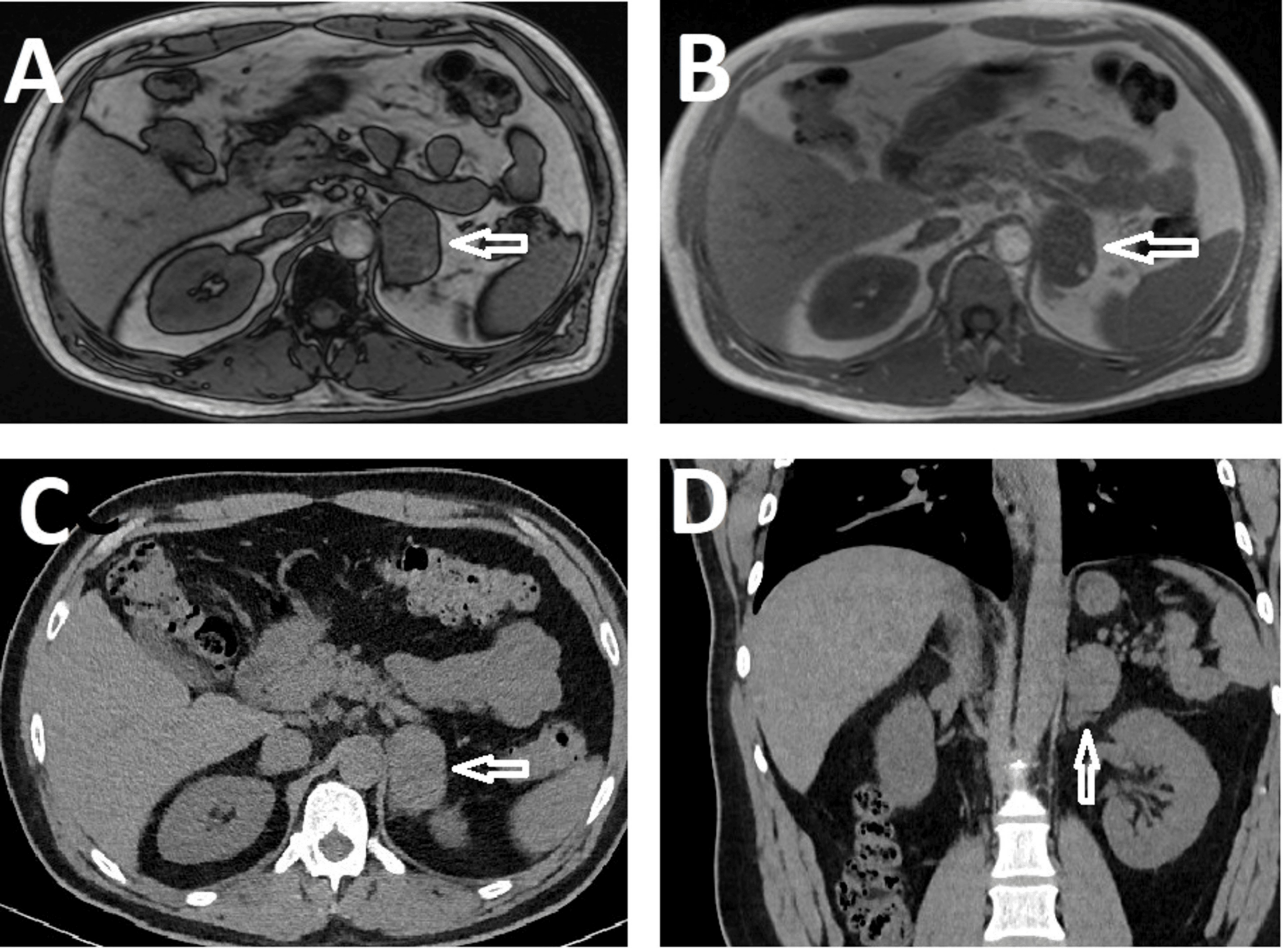
Adrenal imaging A) In-phase axial magnetic resonance imaging (MRI) image of a 4.9 x 3.2 x 4.0 cm ovoid heterogeneous left adrenal mass with internal soft tissue (hollow white arrow). B) Axial MRI image showing no dropout of signal intensity on the opposed, out-of-phase sequence (hollow white arrow). C) Axial computed tomography (CT) scan image. D) A coronal CT scan image of dense, lipid-poor, left adrenal mass with radiodensity measuring 32 Hounsfield units (hollow white arrow).

His PCP ordered laboratory tests and abdominal magnetic resonance imaging (MRI) due to concern for pheochromocytoma. The initial assessment revealed blood pressure of 150/82 mmHg, pulse 80/min, respirations 16/min, weight 84 kg, body mass index (BMI) 30.02 kg/m^2^, and oxygen saturation of 98% on room air. The laboratory workup of the adrenal mass showed plasma epinephrine of 1288 pg/mL (normal level <95 pg/mL), total catecholamines of 1339 pg/mL (normal level <1046 pg/mL), and adrenocorticotropic hormone (ACTH) less than 5 pg/mL (normal range 6-50 pg/mL). An MRI revealed a 4.9 x 3.2 x 4.0 cm left adrenal mass without dropout on the opposed phase and computed tomography (CT) revealed a dense, lipid-poor left adrenal mass with radiodensity of 32 Hounsfield units (Figure 1A-1D).

Based on these results, the patient was referred for further management to endocrinology and endocrine surgery. Further review of patient’s medication list revealed that his recently adjusted antihypertensive regimen included amlodipine 10 mg daily and lisinopril 40 mg daily. He was also taking a 1000 mg supplement of Ashwagandha plant daily for anxiety and insomnia. It has been shown that this supplement can affect the hypothalamic-pituitary-adrenal axis, decreasing ACTH and cortisol levels. He was taken off the supplement pending repeat ACTH and low-dose dexamethasone suppression test (LDDST). Repeat laboratory test results ordered by the surgeon are displayed in Table 1. These tests included a LDDST with adequate suppression. The only abnormal value was the aldosterone/plasma renin activity ratio.

**Table 1:** Repeat laboratory test results ACTH: Adrenocorticotropic Hormone

Laboratory test	Patient’s result	Normal values
Plasma epinephrine (pg/mL)	54	<82
Plasma norepinephrine (pg/mL)	645	199-937
Plasma dopamine (pg/mL)	13	<27
Total plasma catecholamines (pg/mL)	712	<1046
ACTH (pg/mL)	9	6-50
Aldosterone (ng/dL)	14	<28
Plasma renin activity (ng/mL/h)	0.38	0.25-5.82
Aldosterone/plasma renin activity ratio	36.8	0.9-28.9
Free plasma metanephrine (pg/mL)	<25	<=57
Plasma normetanephrine (pg/mL)	102	<=148

However, due to the previously elevated plasma catecholamines, clinical picture, and size of the mass, pheochromocytoma and/or possible malignancy were high on the differential list and left laparoscopic adrenalectomy was scheduled. The site of the possible aldosterone overproduction was uncertain since the radiological features were incompatible with an adrenal adenoma. Additionally, unilateral nonfunctional adrenal tumors are increasingly prevalent in patients over 45 years of age and may be coincidental with any other condition, including primary hyperaldosteronism [5]. On the other hand, the prevalence of bilateral primary hyperaldosteronism is found in up to 30-35% of patients with hypertension and a unilateral adrenal mass [6]. Thus, the only way to confirm the laterality of aldosterone oversecretion in our patient would be via adrenal venous sampling. However, the results of such an invasive diagnostic procedure would not have changed the plan for surgical management, given the size and the radiological features of the left adrenal mass.

**Figure 2:**
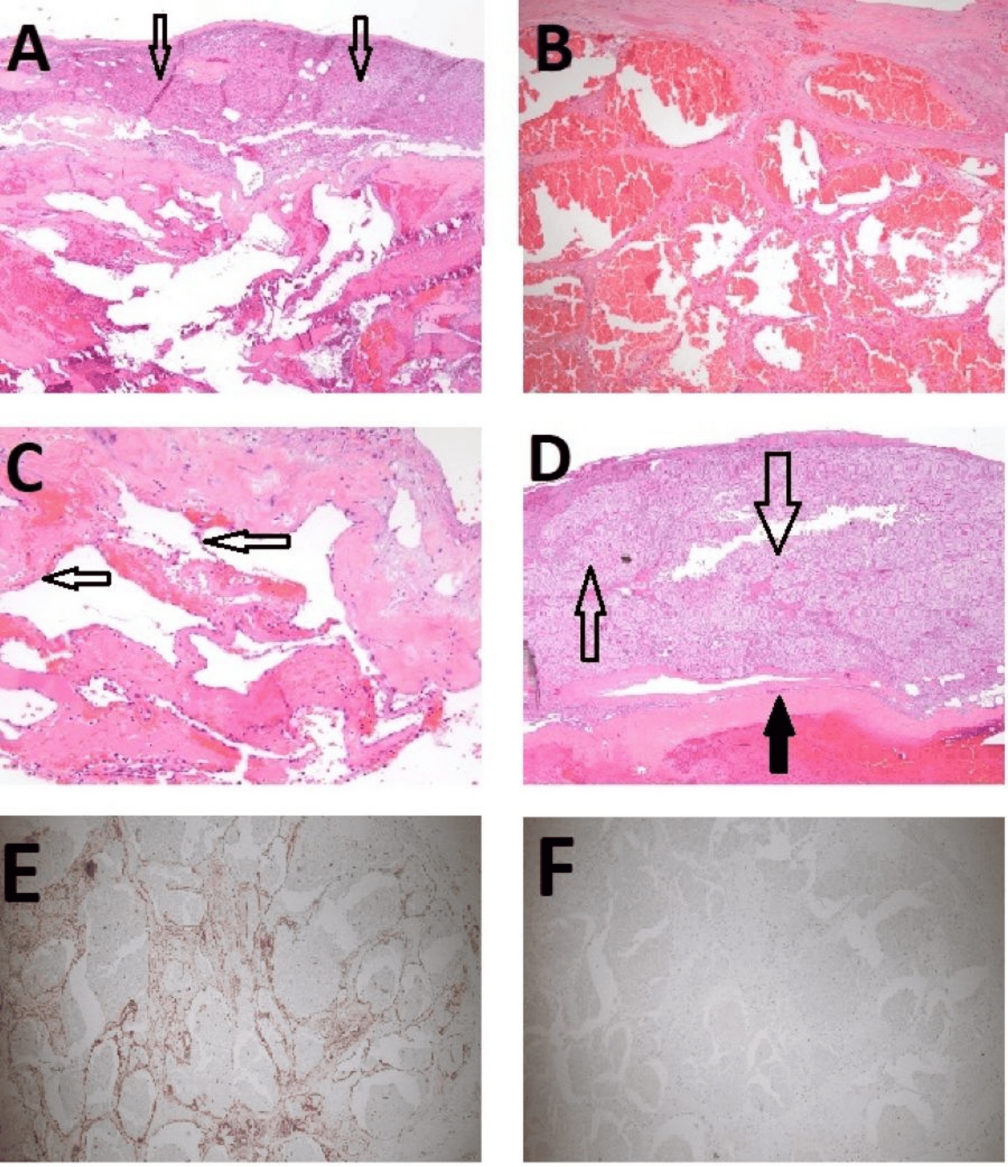
Adrenal histology A) Low-power H&E staining of tumor composed of irregular vascular spaces with fibrotic walls. The overlying cortex is generally atrophic (hollow black arrows). B) Medium-power H&E staining of the tumor composed of irregular vascular spaces. C) High-power H&E staining of the tumor vascular spaces lined by bland endothelial cells (hollow black arrows). D) Low-power view of rare foci of pseudonodular thickened adrenal cortex (hollow black arrows) overlying the fibrotic hemangioma capsule (solid black arrow). E) IHC image showing strong expression of endothelial cell marker CD31 (stained in brown). F) IHC image showing no expression of adenomatoid tumor/mesothelial cell marker calretinin. H&E: Haematoxylin and Eosin; IHC: Immunohistochemical

Pre-operative alpha-blockade was achieved with phenoxybenzamine 10 mg once a day for two weeks prior to surgery, and intravascular volume was optimized with a high salt diet and adequate daily hydration. The surgery was performed in the right decubitus position with three left subcostal trocars technique. The left adrenal gland and the tumor were grossly inseparable and were dissected successfully off the surrounding soft tissues. The surrounding perinephric and periadrenal fat pads were excised to at least one centimeter in depth to ensure clear margins as per oncological principles. The left adrenal gland and the tumor were sent to pathology as a permanent specimen. The patient tolerated the procedure well without intraoperative complications. The pathology report of the adrenal mass revealed a cavernous hemangioma with pseudonodular thickened adrenal cortex (Figures 2A-2D). Immunohistochemical (IHC) staining with endothelial and mesothelial markers was performed to distinguish between a hemangioma and pathologically similar lesions such as adenomatoid tumors. The IHC staining showed strong expression of endothelial cell marker CD31 and no expression of adenomatoid tumor/mesothelial cell marker calretinin, confirming the diagnosis of hemangioma (Figures 2E, 2F). Specific staining for 11-beta-hydroxylase was not performed due to the lack of commercially available antibodies.

The patient was seen as an outpatient one week after the surgery with another follow-up two months later. He was recovering well without complications. His blood pressure therapy was adjusted postoperatively to a lower dose of amlodipine (5 mg daily) and he remained on lisinopril 40 mg daily. He achieved excellent blood pressure control with levels of 120-130/80 mmHg. His aldosterone/plasma renin ratio was checked one year after surgery and had normalized to 3.2 from the preoperative level of 36.8 (normal range 0.9-28.9). The patient's sleep pattern and anxiety had improved as well.

## Discussion

The differential diagnoses for the most frequently encountered adrenal masses include adrenal adenomas, pheochromocytomas, metastatic lesions, adenomatoid tumors (benign neoplasms composed of mesothelial cells), and adrenal cortical carcinomas [7,8]. Cavernous hemangiomas are benign lesions formed from dilated vessels and are commonly located in the liver and the brain [1]. Histological examination reveals unencapsulated masses consisting of stromal connective tissue that separates vascular spaces [1]. They commonly contain calcifications and thromboses [1]. These lesions bleed easily and can lead to hemorrhage, and pseudocysts may form upon resorption of a contained hemorrhage [1,9]. Adrenal cavernous hemangiomas can also cause symptoms from the compression of neighboring structures, resulting in abdominal, flank, or back pain [1,4]. The patient in this case study also presented with left upper flank pain that resolved after adrenalectomy. Given the rarity of adrenal cavernous hemangiomas, the literature deals primarily with case reports. Approximately 70 cases have been published since its first documentation in 1955 [2]. There is no specific blood test for its detection [4]. The majority of adrenal hemangiomas are not associated with hormonal overproduction. However, approximately 10% (eight cases) of patients have been diagnosed with hyperaldosteronism (four cases) and subclinical Cushing syndrome (four cases), as detailed in Table 2 [3,4,10-17].

**Table 2:** Cases with hormone-producing adrenal cavernous hemangiomas

Patient number	Sex	Age (years)	Presentation	Laterality	Size of the tumor (cm)	Hormones secreted	Reference, year
1	M	71	Diaphoresis, fatigue	Left	10 x 18 x 24	Cortisol	Nakagawa, 1986 [10]
2	F	60	Incidental	Right	8	Aldosterone	Stumvoll, 1996 [11]
3	M	59	Incidental	Left	3.1 x 2.9	Aldosterone	Ng, 2008 [12]
4	F	75	Incidental	Left	5 x 5 x 3	Cortisol	Oishi, 2012 [13]
5	M	77	Abdominal pain	Bilateral	10.2 left, 4 right	Cortisol	Lorenzon, 2013 [14]
6	F	78	Incidental	Right	5.4 x 3.3	Cortisol	Edwards, 2017 [15]
7	M	52	Incidental	Left	5 x 3.7 x 3	Aldosterone	Iwamoto, 2018 [16]
8	M	64	Incidental	Left	7.0 x 4.1 x 3.6	Aldosterone	Antar, 2023 [17]

This is among the few reported cases of hemangioma associated with primary hyperaldosteronism that resolved after adrenalectomy. However, the diagnosis of primary hyperaldosteronism and the relationship between the patient’s clinical picture and the aldosterone/plasma renin ratio is at best descriptive and speculative, as there is currently no definitive causative relationship between adrenal cavernous hemangioma and primary hyperaldosteronism [11,12].

The imaging findings are mostly non-specific which makes it difficult to differentiate adrenal hemangiomas from possible malignant lesions, lipid-poor adenomas, or pheochromocytomas. Hemangiomas may present as masses of variable size, echotexture or radiodensity on sonography and CT, respectively [18]. For instance, while lipid-rich adenomas have an unenhanced pathognomonic attenuation on CT of less than 10 Hounsfield units, 30% of adenomas could be lipid-poor and will have an attenuation greater than 10 Hounsfield units, as will pheochromocytomas or malignant masses [18-20]. One of the radiological features of a benign adenoma compared to malignant tumors is the rapid loss of contrast during CT and signal drop off on an out-of-phase MRI [19]. On the other hand, some lipid-poor adenomas, pheochromocytomas, and malignant tumors will retain signal on the out-of-phase MRI imaging, making the radiological diagnosis of hemangiomas challenging [19]. This patient’s adrenal mass had a radiodensity of 32 Hounsfield units on CT and no dropout on the out-of-phase MRI images, which made the biology of the tumor uncertain.

## Conclusions

Our patient’s clinical presentation, tumor size, inconclusive catecholamines and ACTH laboratory studies, and biochemical evidence of primary hyperaldosteronism compounded the diagnostic and management dilemma. Though it was impossible to determine the type of mass until it was surgically removed and assessed by pathology, surgical excision was still the optimal treatment due to the size of the mass and the potential for malignancy and/or pheochromocytoma.

Even though they are rare, adrenal cavernous hemangiomas should be considered in the differential diagnosis of cases where the biochemical and imaging studies are not straightforward and pose diagnostic and management uncertainty.
